# China launches its first diagnostic criteria for occupational post‐traumatic stress disorder

**DOI:** 10.1002/gps3.70024

**Published:** 2026-05-06

**Authors:** Bangshan Liu, Junzhe Cheng, Wei Jiang, Jin Liu, Yumeng Ju, Mi Wang, Gang Wang, Yan Zhang

**Affiliations:** ^1^ Department of Psychiatry National Clinical Research Center for Mental Disorders, and National Center for Mental Disorders The Second Xiangya Hospital of Central South University Changsha Hunan China; ^2^ Mental Health Institute of Central South University China National Technology Institute on Mental Disorders Hunan Technology Institute of Psychiatry Hunan Key Laboratory of Psychiatry and Mental Health Hunan Medical Center for Mental Health Changsha Hunan China; ^3^ Department of Mental Health Center Xiangya Hospital of Central South University Changsha Hunan China; ^4^ The National Clinical Research Centre for Mental Disorders and Beijing Key Laboratory of Mental Disorders and The Advanced Innovation Centre for Human Brain Protection Beijing Anding Hospital School of Mental Health Capital Medical University Beijing China

**Keywords:** post‐traumatic, psychiatry, public policy, stress disorders

## INTRODUCTION

On 1 August 2025, China's GBZ 337–2025 Diagnostic Standard for Occupational Post‐Traumatic Stress Disorder came into force.^1^ For the first time, post‐traumatic stress disorder (PTSD) among emergency responders, including police, firefighters and medical personnel, has been formally recognised as an occupational condition, eligible for compensation under the country's occupational injury system. This move marks a significant institutional acknowledgement that psychological trauma can be as disabling and deserving of protection as physical injury. Far from an isolated policy decision, GBZ 337–2025 marks a milestone in decades of evolving scientific understanding, public awareness and policy development, shaped by the lived experiences of natural disasters and public health emergencies in China.

## THE EVOLUTION OF PTSD AWARENESS IN CHINA

### Conceptual import and early misunderstanding (1980s–1990s)

PTSD was formally introduced to Chinese psychiatry through translations of the 3rd edition of the American Psychiatric Association's Diagnostic and Statistical Manual of Mental Disorders (DSM‐III) during the 1980s–1990s. However, in the absence of a corresponding diagnostic category in the Chinese Classification of Mental Disorders, Second Edition (CCMD‐2), trauma‐related symptoms were commonly conceptualised as ‘delayed psychogenic disorder’ or subsumed under neurasthenia and related stress–response diagnoses.^2^, ^3^ Comparative analyses of CCMD‐2 and Diagnostic and Statistical Manual of Mental Disorders, Third Edition, Revised (DSM‐III‐R) indicate that this nosological mismatch contributed to limited clinical adoption of PTSD beyond tertiary academic centres, with trauma‐related presentations frequently classified under broader stress‐related categories rather than being recognised as a distinct disorder.^4^ Public awareness remained negligible, with most cases subsumed under umbrella categories such as ‘stress‐related disorders’.

### The Wenchuan earthquake and public awakening (2008)

On 12 May 2008, the Wenchuan earthquake in Sichuan province resulted in approximately 70 000 fatalities and displaced over 15 million people. Beyond mobilising massive humanitarian aid, the catastrophe triggered unprecedented media attention to psychological trauma, with coverage documenting distress among survivors, first responders and volunteers with unprecedented granularity. This coverage propelled ‘PTSD’ into public discourse for the first time. Field‐deployed mental health professionals implemented systematic screening and crisis interventions, generating China's first large‐scale epidemiological dataset on trauma‐related disorders.^5^

### Academic and epidemiological expansion (2010s)

The 2010s witnessed the formal integration of PTSD into Chinese nationwide representative epidemiological surveys and academic research. The landmark Chinese Mental Health Survey reported lifetime and 12‐month PTSD prevalence rates of 0.3% and 0.2% in the general population.^6^, ^7^ Notably, prevalence was substantially higher among specific high‐risk groups: survivors of natural disasters, trauma‐exposed children and adolescents and healthcare workers involved in crisis response.^8^, ^9^ For frontline personnel in disaster zones, symptom prevalence reached 20%–30%, aligning with figures reported internationally.^10^, ^11^ Methodologically, research evolved from predominantly cross‐sectional designs towards longitudinal follow‐up studies and the use of culturally adapted assessment instruments.

A key milestone of this era was the publication of the inaugural Chinese Guideline for the Diagnosis and Treatment of Post‐Traumatic Stress Disorder in 2010. This guideline established the first official diagnostic and management framework for PTSD in China, fostering a standardised national approach to trauma‐related mental healthcare. This foundational document underwent a significant update in 2025, incorporating more recent research findings and refining diagnostic criteria and treatment protocols, thereby strengthening China's overall response to PTSD.

### The coronavirus disease 2019 (COVID‐19) pandemic: A national stress test (2019–2022)

The COVID‐19 pandemic represented a prolonged, nationwide exposure to trauma‐like stressors, especially for healthcare workers and public health responders. Extended high‐pressure work, exposure to suffering and death and fear of infection contributed to elevated rates of PTSD and related mental health conditions.^12^ These experiences reinforced the importance of distinguishing occupational PTSD from trauma exposure in the general population, and they accelerated policy discussions about protection and compensation for those in high‐risk roles.

## GBZ 337–2025: POLICY CONTEXT, CONTENT AND LEGAL SIGNIFICANCE

China's GBZ 337–2025 was formulated through collaboration between leading psychiatric hospitals, the Chinese Center for Disease Control and Prevention and other national institutions, reflecting both scientific consensus and interagency coordination. The standard explicitly references internationally recognised diagnostic frameworks (International Statistical Classification of Diseases and Related Health Problems, 10th Revision, ICD‐10). GBZ 337–2025 selectively operationalises core PTSD symptom domains within a legally defined occupational health framework. Table 1 provides a comparison of PTSD diagnostic frameworks in Diagnostic and Statistical Manual of Mental Disorders, Fifth Edition, Text Revision (DSM‐5‐TR), ICD‐10, International Statistical Classification of Diseases and Related Health Problems, 11th Revision (ICD‐11) and GBZ 337–2025.

**TABLE 1:** Comparison of PTSD diagnostic frameworks in DSM‐5‐TR, ICD‐10, ICD‐11 and GBZ 337–2025

Dimension	DSM‐5‐TR (APA, 2022)	ICD‐10 (WHO, 2016)	ICD‐11 (WHO, 2019)	GBZ 337–2025
Official status	Psychiatric diagnostic manual	International Classification of Diseases	International Classification of Diseases	Mandatory national occupational health standard
Target population	General population	General population	General population	Emergency responders and designated rescue workers
Trauma definition	Exposure to actual or threatened death, serious injury or sexual violence (direct, witnessed, indirect or repeated exposure)	An exceptionally threatening or catastrophic event is likely to cause pervasive distress in almost anyone.	Exposure to an extremely threatening or horrific event or series of events	Direct occupational exposure to traumatic incidents occurring in the course of work
Symptom clusters	Four clusters: Intrusion, avoidance, negative alterations in cognition and mood, hyperarousal	Three clusters: Re‐experiencing, avoidance, hyperarousal or inability to recall	Three clusters: Re‐experiencing, avoidance, persistent sense of threat	Three clusters: Re‐experiencing, avoidance, hyperarousal
Symptom breadth	Broad; includes negative mood, guilt, anhedonia, detachment	Moderate; includes emotional numbing and autonomic arousal	Narrow; excludes nonspecific affective and cognitive symptoms	Core PTSD symptoms are required; affective symptoms may be present but are not sufficient alone.
Minimum duration	> 1 month	Symptoms must persist for several weeks, typically > 1 month	Several weeks	> 1 month
Onset specification	No fixed upper limit	Onset usually occurs within 6 months, though a later onset is possible.	No fixed upper limit	Symptoms must emerge within 6 months of the occupational traumatic event.
Functional impairment	Required	Required	Required	Required and explicitly linked to work capacity
Exclusion criteria	Not attributable to substances or medical conditions	Not attributable to organic mental disorder or substance use	Not attributable to substances or medical conditions	Explicit exclusion of other psychiatric disorders, medical conditions or substance‐induced states
Diagnostic purpose	Clinical diagnosis and research	Clinical diagnosis and epidemiology	Clinical diagnosis and epidemiology	Legal determination for occupational injury compensation
Legal applicability	None	None	None	Diagnostic conclusions carry legal effect for work injury insurance.

*Note*: All diagnostic frameworks listed refer to PTSD in its singular form and do not incorporate the extended criteria for cPTSD. For ICD‐11, only the core PTSD symptoms (re‐experiencing, avoidance and sense of threat) are considered herein to facilitate the comparison with other diagnostic frameworks.

Abbreviations: APA, American Psychiatric Association; cPTSD, complex post‐traumatic stress disorder; DSM‐5‐TR, Diagnostic and Statistical Manual of Mental Disorders, Fifth Edition, Text Revision; ICD‐10, International Statistical Classification of Diseases and Related Health Problems, 10th Revision; ICD‐11, International Statistical Classification of Diseases and Related Health Problems, 11th Revision; PTSD, post‐traumatic stress disorder; WHO, World Health Organization.

GBZ 337–2025 specifies that the regulation applies to emergency responders (police, medical personnel, firefighters and other designated rescue workers), whose duties require direct exposure to traumatic incidents. Diagnostic eligibility requires that characteristic symptoms emerge within 6 months of the triggering event and persist for more than 1 month, encompassing the core triad of re‐experiencing, avoidance and hyperarousal. This symptom configuration aligns with the core structure emphasised in ICD‐10 while limiting the diagnostic scope to trauma‐specific manifestations rather than broader affective or cognitive disturbances. Emotional numbing, social detachment or anhedonia may be observed in clinical assessment but are not sufficient as standalone diagnostic criteria.

The diagnostic process mandates a comprehensive evaluation integrating documented occupational history, psychiatric examination and official confirmation of the traumatic incident while systematically excluding alternative explanations such as other medical conditions, psychiatric disorders or substance‐induced states. By embedding diagnostic judgements within a mandatory occupational standard, GBZ 337–2025 prioritises procedural clarity, evidentiary corroboration and functional impairment relevant to work capacity, thereby distinguishing its purpose from that of general psychiatric classification systems.

As a mandatory national standard, GBZ 337–2025 ensures uniform application nationwide, and its diagnostic conclusions carry direct legal weight in determining the status of occupational injuries. By formally linking PTSD to work injury insurance in China, the regulation positions psychological trauma on a par with physical injury in the legal framework of workplace health protection. This legally anchored application underscores the need for diagnostic precision and transparency, particularly in differentiating trauma‐specific pathology from nonspecific psychological distress in compensation determinations. This parity enables affected workers to access compensation, healthcare utilisation and income support, signalling a substantive shift in the legal recognition of mental health in occupational contexts. It lays the groundwork for the future institutionalisation of early screening and intervention for high‐risk professions and the establishment of structured rehabilitation pathways, including return‐to‐work assessments, with the aim of more fully integrating occupational PTSD management into China's developing mental health infrastructure.

## INTERNATIONAL COMPARISONS AND LESSONS

Although PTSD diagnosis is anchored worldwide in standardised clinical frameworks such as DSM‐5 and ICD‐11, the extent to which it is formally recognised as an occupational disease differs markedly across countries. In Canada, several provinces have adopted presumptive legislation for first responders, under which a diagnosis of PTSD in occupations such as firefighting, policing or paramedicine is presumed to be work‐related unless proven otherwise.^13^ This legislative model effectively reduces administrative barriers, expedites access to compensation and fosters the timely initiation of treatment. A similar orientation is evident in Australia, where federal and state schemes, including Comcare, have issued detailed guidance for the assessment of PTSD in emergency service personnel and where certain jurisdictions, such as Queensland, have codified presumptive provisions that remove the need for case‐by‐case causation proof in eligible first responders.^14^ In the United States, the absence of a federal standard has resulted in considerable variation across state workers' compensation systems. Although some states permit compensation for psychological injuries that occur without accompanying physical harm, others have enacted presumptive PTSD statutes specifically for first responders. However, eligibility criteria, evidentiary thresholds and benefit structures vary substantially across jurisdictions, leading to inconsistent access to recognition and compensation.^15^ In the United Kingdom and across the European Union, authoritative clinical guidelines, such as those issued by the National Institute for Health and Care Excellence, provide detailed recommendations on PTSD diagnosis and treatment; however, these guidelines do not guarantee occupational coverage, and compensation is determined on a case‐by‐case basis through legal proceedings that require robust proof of causation.

Against this backdrop, China's GBZ 337–2025 represents a distinct policy model. Rather than adopting presumptive rules that bypass causation requirements, it integrates the diagnostic process directly into a mandatory national occupational health standard. This ensures uniform application of criteria across the country while maintaining strict evidentiary thresholds to confirm work‐relatedness. The Chinese approach seeks to balance the need to protect and compensate affected workers with the imperative of sustaining the occupational injury system's integrity and long‐term viability.

## PERSPECTIVES AND FUTURE DIRECTIONS

The introduction of GBZ 337–2025 represents a watershed moment in China's mental health and occupational safety policy landscape. Its long‐term impact will depend critically on effective implementation. Ensuring adequate diagnostic capacity necessitates systematic training for psychiatrists, occupational physicians and evaluators to guarantee consistent clinical application of the standard. Although the requirement for official documentation of the traumatic event is relatively straightforward in large‐scale disaster contexts, it presents greater complexity in cases involving cumulative or indirect trauma exposures. Furthermore, the current eligibility parameters, which primarily focus on defined categories of emergency responders, may inadvertently exclude other high‐risk occupational groups whose trauma stems from vicarious or secondary exposure. These include not only frontline professionals such as journalists in conflict zones but also responders such as digital forensic investigators and emergency dispatchers, who are chronically exposed to graphic, traumatic material or distressing auditory evidence. To ensure a more inclusive approach, the occupational PTSD eligibility criteria could be expanded to encompass individuals in other high‐risk professions.

Another important challenge concerns the temporal specification requiring symptom onset within 6 months of a documented occupational traumatic event. Although this criterion enhances evidentiary clarity in compensation determinations, it may be less well suited to occupations characterised by prolonged, repetitive or cumulative trauma exposure. For first responders, psychological injury often reflects sustained exposure to distressing material rather than a single, discrete incident, and symptom onset may follow a delayed or gradual trajectory. In addition, prolonged occupational trauma may give rise to more pervasive disturbances in self‐organisation, such as affect dysregulation, negative self‐concept and interpersonal difficulties, which are characteristic of complex PTSD and may not be fully captured by a narrow focus on core PTSD symptoms. Ensuring that the most severely impaired individuals are accurately identified will require careful differentiation between classic PTSD, more complex trauma‐related presentations and general psychological distress, thereby preventing systematic under‐recognition of workers whose functioning has been profoundly altered by chronic trauma exposure.

To ensure that GBZ 337–2025 is translated into valid public health practice, implementation must be grounded in rigorous empirical and psychometric validation rather than reliance on simple symptom counts, which risk conflating trauma‐specific pathology with general occupational distress. Future studies should employ person‐centred analytic approaches, such as latent class analysis, to identify clinically meaningful subgroups and better distinguish PTSD from nonspecific burnout in compensation decisions. Equally critical is establishing measurement equivalence across China's diverse socioeconomic and cultural contexts through confirmatory factor analysis and invariance testing, with priority given to culturally validated instruments (e.g., the International Trauma Questionnaire or other validated measures). In parallel, the operational definition of occupational ‘exposure’ should accommodate cumulative and indirect trauma, supported by longitudinal occupational documentation, corroborative testimony and serial mental health assessments.

From a systems perspective, important structural constraints also warrant consideration. According to the 2024 Statistical Bulletin on Human Resources and Social Security Development, approximately 304 million individuals were covered by work injury insurance in China. This figure represents only part of the broader working‐age population when compared with over 745 million participants in the basic endowment insurance system in 2024. This discrepancy suggests that a considerable proportion of workers may remain outside the scope of occupational injury protection, posing challenges for the equitable implementation of PTSD‐related compensation, particularly for those in informal or flexible employment. On the other hand, the scale of the existing system has significant fiscal implications. In 2024 alone, 2.24 million individuals received work‐related injury insurance benefits, with total annual expenditures approaching 20 billion US dollars. As recognition of occupational PTSD expands, a corresponding increase in claims is likely, raising important questions regarding the capacity and long‐term sustainability of the compensation system. Continuous monitoring of claim patterns, expenditure growth and fund balance will be essential to ensure that policy expansion remains clinically appropriate and economically sustainable.

Beyond China’s borders, GBZ 337–2025 offers valuable lessons, particularly for low‐ and middle‐income countries seeking to strengthen protections for workers in trauma‐exposed professions. Embedding PTSD diagnostic criteria within a mandatory occupational health standard provides a clear and consistent framework for recognition and compensation. Integrating this framework with existing compensation, rehabilitation and mental health service systems promotes policy coherence and operational efficiency. Importantly, such a standard has the potential to encourage upstream preventive measures (psychological preparedness training, resilience‐building initiatives and structured postincident support) that could mitigate the risk of long‐term disability. China’s rich body of disaster epidemiology, spanning earthquakes to epidemics, not only supports the legitimacy of the policy but also offers a data‐driven model for other nations to adapt within their own cultural and institutional contexts.

Ultimately, GBZ 337–2025 should be regarded as a starting point rather than an endpoint. It reflects decades of evolving understanding: from the initial academic introduction of PTSD in China, through public awakening in the aftermath of major disasters, to the development of structured research and ultimately to legal recognition as an occupational injury. To safeguard the long‐term credibility and sustainability of the compensation system, future implementation should prioritise scientifically parsimonious diagnostic frameworks, culturally validated assessment tools and analytic approaches capable of distinguishing trauma‐specific psychopathology from nonspecific occupational distress across diverse socioeconomic settings. As awareness of occupational PTSD grows, an increase in claims is likely. This anticipated increase necessitates close monitoring of fiscal impacts and potential adjustments to eligibility criteria or preventive interventions to maintain the system’s sustainability. For the international community, China’s experience serves as a compelling example of how a nation can move from acknowledging the psychological toll of trauma to institutionalising robust systems of protection. The legal recognition of psychological trauma is more than an administrative measure. It is a declaration of societal values and a recognition that the mind, like the body, bears wounds that merit healing.

## AUTHOR CONTRIBUTIONS

Yan Zhang and Gang Wang conceived the idea for this paper. Bangshan Liu and Junzhe Cheng drafted the original manuscript. Wei Jiang, Jin Liu, Yumeng Ju and Mi Wang revised the manuscript. All authors approved the final version of the manuscript.

## FUNDING

This study was supported by the STI2030‐Major Projects (2021ZD0202000 to Yan Zhang), the Science and Technology Innovation Program of Hunan Province (2023RC3083 to Bangshan Liu) and the Natural Science Foundation of Hunan Province, China (2024JJ2085 to Bangshan Liu and 2026JJ90314 to Junzhe Cheng).

## CONFLICT OF INTEREST STATEMENT

The authors declare no conflicts of interest.

## ETHICAL STATEMENT

Not applicable.

## Data Availability

Data sharing is not applicable to this article as no new data were created or analysed in this study.
